# Postprandial fullness correlates with rapid inflow of gastric content into duodenum but not with chronic gastritis

**DOI:** 10.1186/1471-230X-11-140

**Published:** 2011-12-21

**Authors:** Nobutake Yamamichi, Takeshi Shimamoto, Chihiro Minatsuki, Yoko Yoshida, Mitsuhiro Fujishiro, Shinya Kodashima, Jun Kato, Osamu Goto, Satoshi Ono, Keiko Niimi, Yu Takahashi, Maki Konno-Shimizu, Masao Ichinose, Kazuhiko Koike

**Affiliations:** 1 Department of Gastroenterology, Graduate School of Medicine, University of Tokyo (7-3-1, Hongo, Bunkyo-ku, Tokyo, Japan; 2 Health Department, Human Resources Division, Nippon Steel Corporation (2-6-1, Marunouchi, Chiyoda-ku, Tokyo, Japan; 3 Department of Gastroenterology, School of Medicine, Wakayama Medical University (811-1, Kimiidera, Wakayama-shi, Wakayama, Japan

## Abstract

**Background:**

The aim of this study is evaluating the correlation of postprandial fullness with chronic gastritis or rapid inflow of gastric content into duodenum, based on double-contrast barium X-ray imaging.

**Methods:**

253 healthy subjects who underwent upper gastrointestinal barium X-ray examination were analyzed. Chronic gastritis was judged from mucosal atrophy and hypertrophic thickened folds on barium X-ray images. For the gastric excretion, the tips of barium flow on the single-contrast frontal barium X-ray images of the stomach were classified into four categories; V type (all the barium remained in the stomach), V-H type (some barium had flowed into the duodenum but the tip of barium remained in the proximal half of the duodenal bulb), H-V type (some barium had flowed into the duodenum and the tip of barium was in the distal half of duodenal the bulb, but no barium was observed in the descending part of the duodenum), and H type (some barium had flowed into the descending part of the duodenum). The chi-square test and Cochran-Mantel-Haenzel test were used for evaluation.

**Results:**

Chronic gastritis was observed in 72 subjects, among which 21 subjects (29.2%) presented with postprandial fullness. For the remaining 181 subjects without chronic gastritis, 53 subjects (29.3%) complained of postprandial fullness. There is no significant correlation between chronic gastritis and postprandial fullness (p = 0.973). For the rapid flow of gastric content into duodenum, all the 253 subjects comprised 136 subjects with V type (in the stomach), 40 subjects with V-H type (in the proximal half of the duodenal bulb), 21 subjects with H-V type (in the distal half of the duodenal bulb), and 56 subjects with H type (in the descending part of the duodenum). Postprandial fullness was present in 30 subjects with V type (22.1%), 9 subjects with V-H type (22.5%), 8 subjects with H-V type (38.1%), and 27 subjects with H type (48.2%). There is a distinct correlation between postprandial fullness and gastric barium excretion on barium X-ray imaging (p = 0.002).

**Conclusions:**

Bothersome postprandial fullness correlates with rapid inflow of gastric content into duodenum, but not with chronic gastritis.

## Background

Postprandial fullness is defined as an unpleasant sensation like prolonged persistence of food in the stomach. In the Rome III criteria which classifies functional gastrointestinal disorders (FGIDs) into four categories (i.e., functional dyspepsia, belching disorders, nausea/vomiting disorders, and rumination syndrome in adults), bothersome postprandial fullness is described as a major symptom of functional dyspepsia [1,2]. Postprandial fullness, as well as early satiety, is a popular gastrointestinal symptom which occurs to not only FGID patients but also to healthy subjects. However, in both situations, the underlying mechanisms of postprandial fullness remain unclear.

It can be easily imagined that gastritis may cause unpleasant sensation of postprandial fullness. However, despite the many studies evaluating an association between FGID symptoms and gastritis [3-6], studies focusing on the correlation of postprandial fullness and gastritis have been very few. Marzio *et al*. reported that *Helicobacter pylori *infection is not associated with upper abdominal complaints including postprandial fullness and early satiety [7], and Karamanolis *et al*. also reported that the status of *Helicobacter pylori *infection does not correlate with the symptoms of functional dyspepsia [8]. In our study, not *Helicobacter pylori *infection but chronic gastritis itself was precisely diagnosed on the basis of gastric atrophy and/or hypertrophic thickened folds in double-contrast barium X-ray images of the stomach [9,10]. Based on radiologically accurate diagnosis, we tried to evaluate correlation between gastritis and postprandial fullness.

The sensation of a full stomach (satiety) has been reported to be dependent on the volume of the integrated meal, as well as the caloric content and viscosity of the meal [11]. Even the ingestion of water alone could lead to a sensation of satiety [11,12]. Based on these reports, the sensation of satiety seems likely to correlate positively with residual volume in the stomach. We therefore hypothesized that gastric content emptying might influence the sensation of postprandial fullness. Namely, we speculated that subjects with slow gastric content emptying such as those with gastroptosis (downward displacement of the stomach) would tend to have more gastric residue, and thereby be apt to cause the sensation of postprandial fullness. In our study, noncaloric barium was used for the orally taken content, and injection of a spasmolytic agent was performed to relax the muscles of the gastrointestinal walls. Stimulant effect of caloric content upon gastrointestinal movement [12] or autonomic peristalsis of the stomach could thus be excluded.

Consequently, our study examined the correlation of bothersome postprandial fullness with gastric barium excretion, which is followed by rapid inflow of barium into the duodenum. Although the relative density of barium contrast medium is much heavier than normal food, the result of this study using noncaloric barium should reflect the effect of rapid inflow of gastric content into duodenum to some extent.

## Methods

### Subjects

Subjects comprised 285 individuals (273 men and 12 women) who underwent medical check-ups at the Health Department, Human Resources Division, Nippon Steel Corporation in 2008. All of them underwent upper gastrointestinal double-contrast barium X-ray examination for screening of gastric cancer. For evaluation of serum anti-*H. pylori *antibody, we used a commercial EIA kit called E-plate EIKEN *H. pylori *antibody (Eiken Chemical Co.,LTD., Tokyo, Japan) according to the previous report [13]. This study was approved by the Working Group of Research Ethics, Human Resources Division of Nippon Steel Corporation. All the data were completely anonymized, and were handled carefully according to the Declaration of Helsinki.

### Upper gastrointestinal X-ray examination

Ten minutes after intramuscular injection of spasmolytic agent (7.5 mg of prifinium bromide), the subject drank 150 ml of barium in one gulp. X-ray images were then taken as follows; 1) double-contrast image of the upper and lower esophagus in the right anterior oblique standing position, 2) single-contrast image of the stomach in the frontal standing position, 3) double-contrast image of the stomach in the supine position, 4) single-contrast image of the stomach in the prone position, 5) double-contrast image of the stomach in the right anterior oblique supine position, 6) double-contrast image of the stomach in the prone position, 7) double-contrast image of the stomach in the left anterior oblique supine position, 8) double-contrast image of the stomach in the left anterior oblique half-standing position, and 9) double-contrast image of the stomach in the frontal standing position.

### Symptoms of postprandial fullness

As for evaluation of postprandial fullness, we used the question "Does your stomach feel heavy after meals?" from Frequency Scale for the Symptoms of GERD (FSSG) [14]. According to the answer to this question administered prior to the upper gastrointestinal barium X-ray examination, the subjects were classified into two groups: postprandial fullness-positive (+) and postprandial fullness-negative (-).

### Evaluation of chronic gastritis

**Figure 1:**
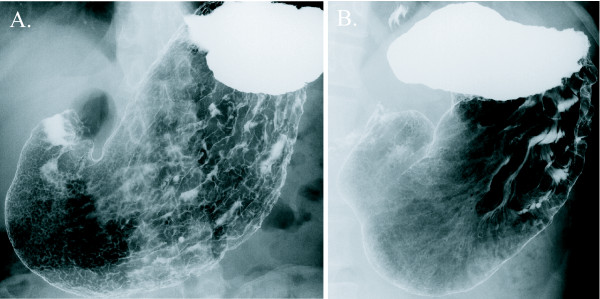
**The typical two types of chronic gastritis observed in the double-contrast barium X-ray images**. (A) Atrophic gastritis with enlarged areae gastricae. (B) Hypertrophic gastritis with thickened folds on the greater curvature.

Chronic gastritis was defined as gastric atrophy with enlarged areae gastricae or hypertrophic gastritis with thickened folds on the greater curvature [9]. Both atrophic and hypertrophic gastritis were typically observed in the stomach with chronic *H. pylori *infection [9,10], and were precisely evaluated based on the double-contrast stomach images on the barium X-ray examination (Figure 1).

### Evaluation of rapid inflow of gastric barium into duodenum on upper gastrointestinal X-ray examination

**Figure 2:**
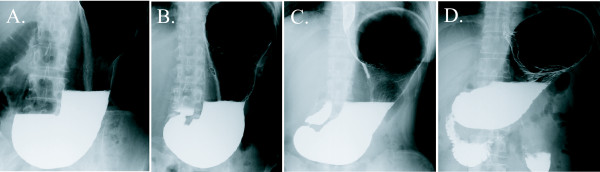
**The degrees of gastric barium excretion based on the tip of barium flow in a single-contrast frontal view of the stomach in the standing position**. (A) V type (in the stomach). (B) V-H type (in the proximal half of the duodenal bulb). (C) H-V type (in the distal half of the duodenal bulb). (D) H type (in the descending part of the duodenum).

Based on the flow of barium during the examination, all subjects were classified into one of the following four categories: V, V-H, H-V, and H type. Soon after the subject drank 150 ml of barium, the single-contrast and frontal X-ray image was taken in the standing position. At this moment, the tips of barium flow were classified as follows (Figure 2);

V type (in the stomach): all the barium remained in the stomach (i.e., no barium had flowed into the duodenum).

V-H type (in the proximal half of the duodenal bulb): some barium had flowed into the duodenum, but the tip of barium remained in the proximal half of the duodenal bulb.

H-V type (in the distal half of the duodenal bulb): some barium had flowed into the duodenum and the tip of barium was in the distal half of duodenal the bulb, but no barium was observed in the descending part of the duodenum.

H type (in the descending part of the duodenum): some barium had flowed into the descending part of the duodenum.

### Statistical methods

We used JMP software (SAS Institute, NC, USA) for statistical analyses. We did not separate the sexes, since the sample size of women was too small. Correlations between age and categorical variables were compared using the χ^2 ^test and Fisher's exact test. To evaluate relationships between postprandial fullness and gastric barium excretion or chronic gastritis on barium X-ray images, the χ^2 ^test, Fisher's exact test, and Cochran-Mantel-Haenszel test adjusted for age were used. Two-sided p-values <0.05 were considered as statistically significant.

## Results

### Characteristics of the study subjects

Of the 285 subjects, we excluded 30 subjects with poor X-ray images (1 subject with previous gastric resection, 26 subjects with uncontrollable spastic movement of stomach, 2 subjects with massive gastric residue, and 1 subject with inadequate position change due to hemiplegia) and 2 subjects with insufficient information obtained from the questionnaire. Mean age for the remaining 253 subjects (241 men and 12 women) was 48.6 ± 7.04 years (range, 32-69 years).

### Validation and age distribution of chronic gastritis

To validate our diagnosis of chronic gastritis judged from gastric atrophy and/or hypertrophic thickened folds (Figure 1), we analyzed another set of 30 subjects, who were tested positive for serum antibody against *H. pylori*. Among the 30 subjects with *H. pylori *infection, 29 subjects (97%) were diagnosed as chronic gastritis based on the double-contrast barium X-ray images. We therefore concluded that our diagnosis of atrophic and/or hypertrophic gastritis certainly reflects the chronic *H. pylori *infection.

Age distribution of subjects with chronic gastritis is shown in Table 1, which indicates a significant correlation between age and chronic gastritis (p = 0.005). It probably reflects the prevalence of *H. pylori *infection in Japanese population; older age is related to *H. pylori *infection and atrophic gastritis [15,16]. In contrast, age and postprandial fullness shows no significant correlation (p = 0.395; Table 2).

**Table 1:** Association of age groups and chronic gastritis.

	Chronic gastritis					
				
	Total (n = 253)	(+)	(-)			
				
	n (%)	n (%)	n (%)	OR (95% CI)	χ^2^	*P*-value
Age (years)						
<39	13 (5.1)	1 (1.4)	12 (6.6)	reference		
40-44	67 (26.5)	12 (16.7)	55 (30.4)	2.618 (0.310-22.108)		
45-49	58 (22.9)	16 (22.2)	42 (23.2)	4.571 (0.549-38.075)		
50-54	67 (26.5)	22 (30.6)	45 (24.9)	5.867 (0.716-48.042)		
55-59	30 (11.9)	10 (13.9)	20 (11.0)	6.000 (0.680-52.902)		
60<	18 (7.1)	11 (15.3)	7 (3.9)	18.857 (1.989-178.803)	16.844	0.005

χ^2 ^test.

**Table 2:** Characteristics of study subjects stratified by the sensation of postprandial fullness.

	Postprandial fullness					
				
	Total (n = 253)	(+)	(-)			
				
	n (%)	n (%)	n (%)	OR (95% CI)	χ^2^	*P*-value
Age (years)						
<39	13 (5.1)	4 (5.4)	9 (5.0)	reference		
40-44	67 (26.5)	15 (20.3)	52 (29.1)	0.649 (0.175-2.406)		
45-49	58 (22.9)	20 (27.0)	38 (21.2)	1.184 (0.324-4.329)		
50-54	67 (26.5)	24 (32.4)	43 (24.0)	1.256 (0.349-4.514)		
55-59	30 (11.9)	8 (10.8)	22 (12.3)	0.818 (0.196-3.416)		
60<	18 (7.1)	3 (4.1)	15 (8.4)	0.450 (0.081-2.488)	5.178	0.395†
Chronic gastritis						
(+)	72 (28.5)	21 (28.4)	51 (28.5)	reference		
(-)	181 (71.5)	53 (71.6)	128 (71.5)	1.006 (0.552-1.833)	0.001	0.973† †
Gastric barium excretion						
V	136 (53.8)	30 (40.5)	106 (59.2)	reference		
V-H	40 (15.8)	9 (12.2)	31 (17.3)	1.026 (0.440-2.390)		
H-V	21 (8.3)	8 (10.8)	13 (7.3)	2.174 (0.825-5.733)		
H	56 (22.1)	27 (36.5)	29 (16.2)	3.290 (1.696-6.381)	14.806	0.002†

† χ^2 ^test.

† † Cochran-Mantel-Haenszel test adjusted for age.

### No correlation exists between chronic gastritis and postprandial fullness

Among all the 253 subjects, 72 subjects (28.5%) were diagnosed as chronic gastritis and 181 subjects (71.5%) had normal gastric mucosa. For the 72 subjects with chronic gastritis, 21 subjects (29.2%) reported postprandial fullness. For the 181 subjects without chronic gastritis, 53 subjects (29.3%) complained of postprandial fullness (Table 2). The χ^2 ^test and Cochran-Mantel-Haenszel test yielded p-values of 0.986 (odds ratio (OR), 1.006; 95% confidential interval (CI), 0.552-1.833) and 0.973 (OR, 0.989; 95% CI, 0.316-3.100) respectively, showing no significant correlation between chronic gastritis and postprandial fullness.

### An obvious correlation exists between rapid inflow of gastric content into duodenum and postprandial fullness

Judged from rapid inflow of gastric content into duodenum on the upper gastrointestinal X-ray imaging, all subjects were divided into four categories; V, V-H, H-V, and H type (Figure 2). All the 253 subjects comprised 136 subjects with V type (53.8%), 40 subjects with V-H type (15.8%), 21 subjects with H-V type (8.3%), and 56 subjects with H type (22.1%) (Table 2). The sensation of postprandial fullness was present in 30 of the 136 subjects with V type (22.1%), 9 of the 40 subjects with V-H type (22.5%), 8 of the 21 subjects with H-V type (38.1%), and 27 of the 56 subjects with H type (48.2%). The χ^2 ^test yielded a p-value of 0.002 (OR, 3.290; 95% CI, 1.696-6.381), showing a distinct correlation between postprandial fullness and rapid inflow of gastric barium into duodenum (Table 2). This result strongly suggests that bothersome postprandial fullness is prone to be present in situations where intragastric content rapidly flows into the duodenum.

## Discussion

In this study using the upper gastrointestinal barium X-ray images, we were unable to detect any relationship between postprandial fullness and chronic gastritis (atrophic and/or hypertrophic gastritis). Our result is consistent with previous reports, which denied the relation of *H. pylori *infection to postprandial fullness and early satiety [7,8]. Given the validation that our diagnosis of chronic gastritis mostly reflected the *H. pylori *infection, we concluded that no association exists between postprandial fullness and chronic gastritis.

Our novel finding was that subjects with rapid inflow of gastric content into duodenum have significant correlation with consciousness of postprandial fullness (Table 2). Counter to our expectations, the sensation of postprandial fullness does not tend to occur in subjects categorized in V type, where most of the subjects with gastroptosis are included. On the contrary, postprandial fullness tends to be present in situation where intragastric content flows rapidly into the duodenum. Based on these results, we speculate that the gastroduodenal shape should be one of crucial factors for postprandial fullness, as gastric barium excretion considerably depends on the shapes of stomach and duodenum.

Previous studies have reported that satiety signals arise from the stomach [17], and are transmitted to the central nervous system through vagal and/or splanchnic nerves via chemoreceptors and mechanoreceptors in the gastric wall [18,19]. Several reports have also suggested that pressure on gastric wall may induce dysplastic symptoms including satiation [20-22]. However, these reported mechanisms do not seem applicable to our results, as there should be no difference in effects on gastric wall among four types of gastroduodenal shapes, which represents four types of gastric barium excretion (V, V-H, H-V, and H type in Figure 2). Although the mechanisms underlying postprandial fullness remain unclear, we speculate that some mechanical or chemical stimuli affecting intestinal wall (duodenum and/or small intestine) may play critical roles in causing the sensation of postprandial fullness.

A recent study reported that higher body mass index (BMI) as well as gastric volume during fasting is clearly associated with reduced satiety [23]. A larger scale study including BMI should thus be performed, as there might be some correlation between gastric shape and BMI. In the near future, we are planning to take a large cross-sectional study of about 7,000 healthy subjects, in which not only chronic gastritis and gastric barium excretion but also BMI, age, sex, serum anti-*H. pylori *IgG, serum pepsinogen I and II levels [24], smoking habit, alcohol drinking, etc. will be evaluated. Our novel finding will be strictly verified in the forthcoming larger scale study based on multivariate analyses. At long last, some animal model should be also necessary to validate the correlation between rapid inflow of gastric content into duodenum and the sensation of postprandial fullness.

## Conclusion

There is a distinct correlation between postprandial fullness and gastric barium excretion on upper gastrointestinal X-ray imaging: bothersome postprandial fullness is prone to be present in situations where intragastric content rapidly flows into the duodenum. On the contrary, no correlation exists between postprandial fullness and chronic gastritis judged from mucosal atrophy and hypertrophic thickened folds on barium X-ray images.

## Competing interests

The authors declare that they have no competing interests.

## Authors' contributions

Author's contributions were as follows: NY (study concept and design, acquisition of data, analysis and interpretation of data, statistical analysis, drafting of the manuscript), TS (analysis and interpretation of data, statistical analysis), CM (analysis and interpretation of data), YY (acquisition of data), MF (administrative support), SK (acquisition of data), JK (analysis and interpretation of data), OG (acquisition of data), SO (acquisition of data), KN (acquisition of data), YT (analysis and interpretation of data), MK (acquisition of data), MI (critical revision of the manuscript for important intellectual content, drafting of the manuscript), and KK (study supervision). The final manuscript has been carefully read and approved by all the authors.

## Pre-publication history

The pre-publication history for this paper can be accessed here:

http://www.biomedcentral.com/1471-230X/11/140/prepub
